# Oral Administration of Fermented Soymilk Products Protects the Skin of Hairless Mice against Ultraviolet Damage

**DOI:** 10.3390/nu8080514

**Published:** 2016-08-20

**Authors:** Mitsuyoshi Kano, Norihiro Kubota, Norie Masuoka, Tetsuji Hori, Kouji Miyazaki, Fumiyasu Ishikawa

**Affiliations:** Yakult Central Institute, Tokyo 186-8650, Japan; norihiro-kubotal@yakult.com.jp (N.K.); norie-masuoka@yakult.co.jp (N.M.); tetsuji-hori@yakult.co.jp (T.H.); koji-miyazaki@yakult.co.jp (K.M.); fumiyasu-ishikawa@yakult.co.jp (F.I.)

**Keywords:** ultraviolet radiation, oxidative DNA damage, skin thickness, isoflavone, soymilk, fermented soymilk

## Abstract

The protective effect of isoflavones on skin damage from ultraviolet (UV) radiation and their bioavailability were investigated in ovariectomized hairless mice fed diets composed of fermented soymilk containing aglycone forms of isoflavones or control soymilk containing glucose-conjugated forms of isoflavones. The erythema intensity of dorsal skin was significantly higher in ovariectomized mice than in sham-operated mice (*p <* 0.05). The erythema intensity and epidermal thickness of dorsal skin were significantly lower in the fermented soymilk diet group than in the control diet group (each *p <* 0.05). Levels of cyclobutane pyrimidine dimers in dorsal skin were significantly lower in the fermented soymilk diet group than in the control group (*p <* 0.05). Serum and dorsal skin isoflavone concentrations were significantly higher in the fermented soymilk diet group than in the soymilk diet group (*p <* 0.05). These results indicate that oral administration of a fermented soymilk diet increases isoflavone concentrations in the blood and skin, effectively scavenging the reactive oxygen species generated by UV irradiation and exerting an estrogen-like activity, with a consequent protective effect on skin photodamage in hairless mice.

## 1. Introduction

Ultraviolet (UV) irradiation has various harmful effects, including skin inflammation, thickening, and cancer [1,2]. In recent years, increased UV exposure from the destruction of the ozone layer and lifestyle changes entailing more outdoor activities has been a concern as these factors increase the incidence of skin cancer [3,4,5]. The incidence of skin cancer has increased in recent decades. In particular, skin cancer is a serious problem in the United States. It is estimated that over 1 million new cases of non-melanoma skin cancer occur each year and that 20% of Americans will be affected by skin cancer in their lifetime [4,5,6].

Skin cancers such as basal cell carcinomas and squamous cell carcinomas, although relatively curable, cause significant cosmetic, physical, and psychological suffering to patients and represent a substantial economic burden on healthcare systems. Malignant melanoma is relatively rare but among the most fatal of all types of cancer.

There are different strategies for protecting the skin against damage from UV irradiation. For general protection, these strategies include avoidance of sun exposure, wearing protective clothing, and topical application of sunscreens containing UV-absorbing constituents, such as titanium dioxide and zinc oxide. Recently, there have been reports of the photoprotection provided by topical and oral administration of vitamins, carotenoids, and flavonoids. Dietary supplementation with vitamins, minerals, or essential fatty acids improves the skin condition of animals, with nutrients such as vitamins A, E, and C and herbal extracts such as green tea protecting the skin against photodamage [7,8,9]. In addition, isoflavones (genistein and equol) inhibit the erythema and DNA damage caused by UV irradiation [10,11,12,13].

Thus, an inhibitory effect on the damage caused by UV irradiation has been reported for various food ingredients. However, many of these reports concern topical application and there have been few studies of the effects of the oral administration of food constituents. To obtain photoprotective effects from oral administration, it is important that the active ingredient reaches a sufficient concentration at the target site. Accordingly, examination of the bioavailability of the active ingredient is required.

Isoflavones are non-steroidal phytoestrogenic and antioxidative polyphenolic molecules with the potential to protect against hormone-dependent conditions, such as breast cancer, prostate cancer, menopausal symptoms, cardiovascular disease, and osteoporosis [14,15,16,17,18,19]. The natural isoflavones in soybeans and unfermented soyfoods occur as glucose-conjugated forms [20]. Once ingested, isoflavone glucosides are hydrolyzed to absorbable aglycones [21]. Intestinal microflora affect the metabolism or absorption of isoflavones as, for example, when they are hydrolyzed to aglycones or transformed into metabolites such as equol or *O*-desmethylangolensin (*O*-DMA) from daidzein [22,23]. Previously, we showed that isoflavone aglycones are absorbed quickly compared with glucoside forms [24,25].

Soy products, which are a rich source of isoflavones, have attracted much attention for their potential health benefits. In particular, soymilk is the main ingredient for making other soy products, and is also an excellent beverage in itself that has been widely consumed in Japan. Recently, fermented soymilk, which is fermented by lactic acid bacteria and bifidobacteria, is also becoming common.

In this study, we investigated the effects of the oral administration of two types of soymilk products—isoflavone glucoside-rich soymilk and aglycone-rich fermented soymilk (FSM)—on UV radiation-induced skin inflammation and isoflavone bioavailability.

## 2. Materials and Methods

### 2.1. Preliminary UVB Irradiation Experiment

Twenty female hairless mice (SKH-hr1) aged 11 weeks were purchased from Japan SLC (Shizuoka, Japan) and housed in cages in a room with controlled lighting (lights on from 08.30 to 20.30 h), temperature (24 ± 2 °C), and humidity (60% ± 5%). Mice were ovariectomized (OVX) or underwent sham operation (Sham) (*n* = 10 each) and were given free access to AIN-93G purified diets [26] and distilled water. They were maintained and treated in accordance with the guidelines of the Ethical Committee for Animal Experiments of Yakult Central Institute. After a 7-day adaptation period, OVX and Sham mice were separated into two groups, the UVB irradiation group and the UVB non-irradiated group, respectively (*n* = 5 each). Mice were irradiated (52 mJ/cm^2^) in the dorsal area. After 5 days, dorsal skin color was evaluated using a chromatometer.

### 2.2. Main UVB Irradiation Experiments

#### 2.2.1. Preparation of FSM

Crude soymilk from Shikokukakouki (Tokushima, Japan) was used as the starting material for FSM. *Bifidobacterium breve* strain Yakult and *Lactobacillus mali* YIT 0243 were obtained from the Culture Collection Research Laboratory of Yakult Central Institute (Tokyo, Japan). A seed preculture prepared anaerobically in the soymilk was freshly added to autoclaved (121 °C, 15 min) soymilk at a 1:100 inoculation ratio and fermented statically at 37 °C for 21 h. The titratable acidity, pH, and viable cell count of the FSM were 0.645%, 4.8, and 1.53 × 10^12^ (*B. breve*) and 1.26 × 10^12^ (*L. mali*) colony-forming units/L, respectively. The original soymilk and FSM were freeze-dried and milled until the products passed through a 0.84-mm sieve (20-mesh). The crude protein, crude fat, ash, and sugar contents in the soymilk were 4.5%, 3.2%, 0.7%, and 2.8%, respectively. This composition was unaffected by the fermentation process, as described previously [27]. The isoflavones present in soymilk and FSM are listed in Table S1. The proportion of aglycones in soymilk and FSM were <1% and >90%, respectively. Compositions of the experimental diets are listed in Table S2.

#### 2.2.2. Mice

**Figure 1:**
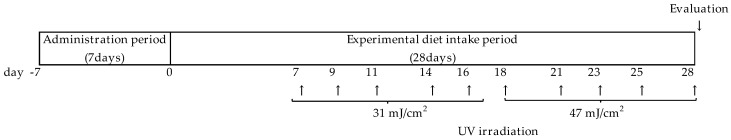
Treatment scheme for main UVB irradiation experiments.

Twenty-four OVX female SKH-hr1 mice aged 7 weeks were purchased from Japan SLC and housed as in the preliminary UVB irradiation experiments. The treatment scheme is shown in Figure 1. After a 7-day adaptation period, mice were randomly divided into four groups (*n* = 6 each) and received the control diet without UV irradiation (untreated group), control diet with UVB irradiation (control group), soymilk diet with UVB irradiation (SM group), and FSM diet with UVB irradiation (FSM group) for 28 days. Seven days after administration of the experimental diet, mice were exposed three times weekly to five UVB sessions of 31 mJ/cm^2^ (from the first to the fifth) and five UVB sessions of 47 mJ/cm^2^ (from the sixth to the tenth). Mice were sacrificed under anesthesia 3 h after the last dose of UVB irradiation. Blood samples were collected from the postcaval vein, and serum was obtained by centrifugation at 2000*× g* for 20 min at 4 °C. Serum was stored at −70 °C until required for analysis.

#### 2.2.3. UV Irradiation

Hairless mice were placed in a plastic cage (W 172 mm × D 240 mm × H 129 mm) and irradiated using a bank of five unfiltered fluorescent sun lamps (FL20SE, Toshiba Leitec, Tokyo, Japan). The distance from the lamps to the dorsal area of the mice was 16 cm. The intensity of UVB irradiation was measured using a VLX-3 W radiometer with a CX-312 sensor (Vilber Lourmat, Marne La Vallee, France).

#### 2.2.4. Erythema and Skin Thickness

Three hours after the last UVB irradiation, dorsal skin color was measured using a chromatometer (CM-2600d; Konica Minolta, Tokyo, Japan) with a three-dimensional color system (L, a, and b values); the a-value (red-green axis) was used to evaluate reddening (erythema). Dorsal skin samples (about 5 cm^2^) were removed after the mice were sacrificed under anesthesia. The skin samples were embedded using TISSU MOUNT (Chiba Medical, Tokyo, Japan) and stored in frozen blocks at −70 °C. Cryostat sections (4 μm thick) were cut from the frozen blocks. The cryostat sections were fixed in 10% buffered formalin and stained with hematoxylin and eosin for routine histological examination.

Skin-fold thickness was measured in the dorsal skin samples using a digimatic indicator (Mitutoyo Corporation, Kanagawa, Japan). Epidermal thickness was measured in the hematoxylin and eosin-stained samples. Cross-sections were selected from three plates per sample and two different microscopic fields per plate were photographed. The graders were blinded to the radiation dose received by the mice.

#### 2.2.5. Enzyme-Linked Immunosorbent Assay for Mouse IL-6

The endogenous concentration of the cytokine IL-6 was determined in mouse serum using a CytoSet mouse IL-6 enzyme-linked immunosorbent assay (ELISA) kit (Biosource International, Camarillo, CA, USA).

#### 2.2.6. Preparation of Skin Samples

For the biochemical analysis, dorsal skin samples frozen in liquid nitrogen were crushed using a CRYO-PRESS^®^ (CP-100 W; Microtec Nition, Tokyo, Japan). Samples of powdered skin were stored at −70 °C until required for analysis.

#### 2.2.7. Thymine Dimer Analysis

The DNA photoproducts cyclobutane pyrimidine dimer (CPD) and pyrimidine-pyrimidone photoproducts (6-4PPs) were assayed according to the method of Takahashi et al. [28]. DNA was purified with a QIAamp DNA Mini Kit (QIAGEN, Hilden, Germany) from samples of frozen skin powder. The DNA concentration was calculated from the absorbance at 260 nm, and the CPD and 6-4PP levels in genomic DNA were determined by ELISA with monoclonal antibodies for these photoproducts in DNA (TDM-1 for CPD and 64M-2 for 6-4PP). Next, 96-well polyvinylchloride flat-bottom microtiter plates (Dynatech, Chantilly, VA, USA) pre-coated with 1% protamine sulfate (Wako, Osaka, Japan) were coated with sample DNA (15 ng per well for CPD and 300 ng per well for 6-4PP). The binding of monoclonal antibodies to photoproducts was detected in immobilized DNA in wells (in quadruplicate) by the biotinylated F (ab’) 2 fragment of goat anti-mouse immunoglobulin G (Zymed, San Francisco, CA, USA) and streptavidin-peroxidase (Invitrogen, Carlsbad, CA, USA). The absorbance of the colored products derived from o-phenylene diamine (Nacalai Tesque, Kyoto, Japan) was measured at 492 nm.

#### 2.2.8. Lipid Peroxide and Thiobarbituric Acid-Reactive Substance Analysis

Lipid peroxide (LPO) concentration in serum was determined using a Determiner LPO kit (Kyowa Medics, Tokyo, Japan).

The level of thiobarbituric acid-reactive substances (TBARS) in the skin homogenate was measured according to the method of Kikugawa et al. [29]. The frozen skin powder was homogenized in a 9-fold volume of 1.15% KCl on ice. Next, 0.1 mL of the skin homogenate was heated at 100 °C for 60 min in 0.2 mL of 0.8% sodium dodecyl sulfate, 1.5 mL of 20% acetic acid buffer (pH 3.5), 50 mL of 0.8% butylated hydroxytoluene, 1.5 mL of 0.8% thiobarbituric acid solution, and 0.7 mL distilled water. After cooling in ice water, TBARS were extracted with 1 mL distilled water and 4 mL n-butanol:pyridine (15:1, *v/v*), followed by centrifugation at 900*× g* for 10 min. The concentration of TBARS was calculated based on the absorbency determined by a spectrometer at 532 nm and the final result was expressed as micromole of TBARS per gram of skin.

#### 2.2.9. Measurement of Isoflavone Concentrations

For the analysis of total isoflavone (conjugated and unconjugated forms), 50 μL of serum or 50 mg of frozen skin powder was added to 50 μL acetate buffer (0.1 mol/L, pH 5.0) containing 100 units of β-glucuronidase and incubated for 15 h at 37 °C to release the aglycone forms of isoflavones from the glucuronide and sulfate conjugates. Both types of samples were then treated with an acetate buffer without β-glucuronidase to analyze unconjugated isoflavone.

Methanol (200 μL) was added to these mixtures, mixed by vortexing and sonication, and then centrifuged at 10,000*× g* for 15 min at 4 °C. The supernatant fluid was filtered through an Ultrafree-MC 0.45 μm and sonication (Merck Millipore, Darmstadt, Germany). A portion was subjected to HPLC. Liquid chromatography-mass spectrometry and HPLC were performed as previously described [24].

#### 2.2.10. Statistical Analysis

All data are expressed as means ± SD. The data were statistically analyzed using SAS version 5.0 (SAS Institute Japan, Tokyo, Japan) by ANOVA and subsequently Tukey’s test for the four groups and unpaired *t*-test for two groups. Differences were considered significant at *p <* 0.05. Power calculation was conducted using SAS version 5.0, and statistical power was more than 80%. 

## 3. Results

### 3.1. Preliminary UVB Irradiation Experiment

#### Effects of Ovariectomy on Erythema

**Figure 2:**
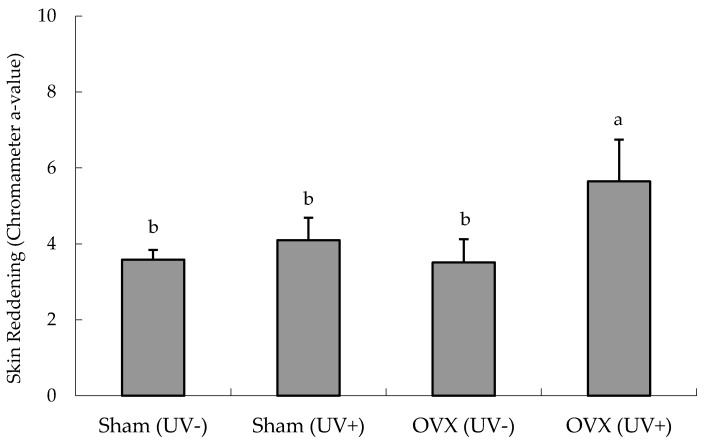
Ultraviolet (UV) B-induced skin reddening (erythema) in ovariectomized hairless mice. The dorsal skin color was measured with a chromatometer five days after the last dose of UVB irradiation and the a-value was used to evaluate erythema. Values are means ± SD (*n* = 5). ^a,b^ Mean values not sharing the same letter above the bars are significantly different (*p <* 0.05, Tukey’s test). Abbreviations: sham, sham-operated mice; OVX, ovariectomized mice.

The effect of the ovariectomy on erythema in the dorsal skin of hairless mice was examined (Figure 2). In the Sham group, there was no difference between UV irradiation and non-irradiation. In the OVX group, erythema was noticeable in the dorsal skin five days after the initial UVB irradiation, and the a-value was significantly increased by UVB irradiation.

### 3.2. Main UVB Irradiation Experiments

Based on the results of Experiment 1—erythema observed in OVX mice—we investigated the effectiveness of isoflavone in these mice.

#### 3.2.1. Effects of Soymilk Products on Photodamage

**Figure 3:**
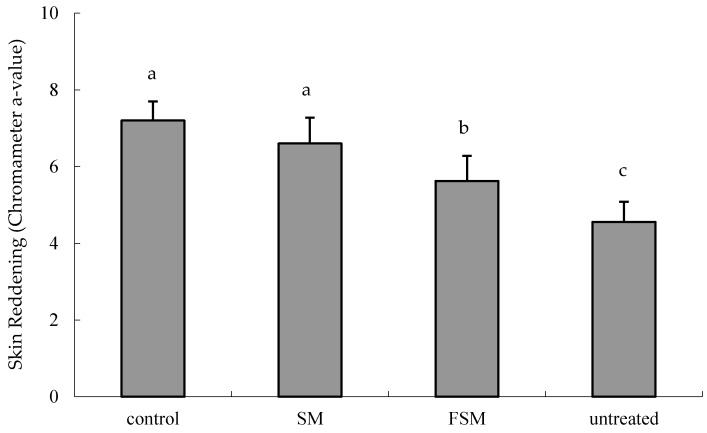
Reduction in ultraviolet (UV) B-induced skin reddening (erythema) by the fermented soymilk diet. The dorsal skin color was measured with a chromatometer 3 h after the last dose of UVB irradiation and the a-value was used to evaluate erythema. Values are means ± SD (*n* = 6). ^a,b,c^ Mean values not sharing the same letter above the bars are significantly different (*p <* 0.05, Tukey’s test). Abbreviations: SM, soymilk; FSM, fermented soymilk.

Body weight and food intake did not differ among the groups (Table S3). The a-value of the dorsal skin was significantly increased by UVB irradiation (*p <* 0.0001, untreated group vs. control group), but was significantly lower in the FSM group than in the control and SM groups (*p <* 0.001 and *p =* 0.0044, respectively) (Figure 3).

**Figure 4:**
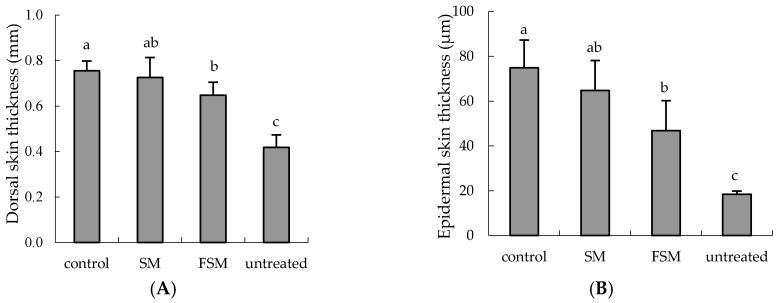

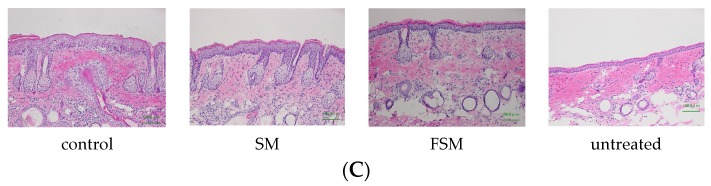
Reduction in ultraviolet (UV) B-induced skin thickness by the fermented soymilk diet. (**A**) effects of UV irradiation on dorsal skin thickness; (**B**) effects of UV irradiation on epidermal skin thickness; (**C**) hematoxylin & eosin staining of UV-irradiated mouse skin. The dorsal area was UV irradiated 10 times; 3 h after the last UV dose, dorsal skin samples were removed and thickness was measured. Values are means ± SD (*n* = 6). ^a,b,c^ Mean values not sharing the same letter above the bars are significantly different (*p <* 0.05, Tukey’s test). Abbreviations: SM, soymilk; FSM, fermented soymilk.

Skin-fold and epidermal thickness were significantly increased by UVB irradiation (*p <* 0.0001, untreated group vs. control group). Skin-fold thickness was significantly lower in the FSM group than in the control group (*p =* 0.0375) (Figure 4A). Epidermal thickness was significantly lower in the FSM group than in the control group (*p =* 0.0019) and tended to be lower than in the SM group (*p =* 0.0554) (Figure 4B). Figure 4C shows representative images indicating that the UVB irradiation increased the epidermal thickness of hairless mice and that FSM administration prevented this increase.

**Figure 5:**
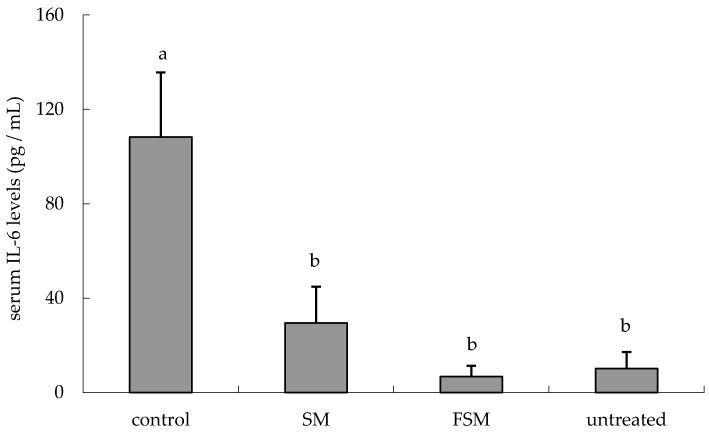
Reduction in ultraviolet (UV) B-induced serum IL-6 levels according to the soymilk and fermented soymilk diets. The dorsal area was UV irradiated 10 times; 3 h after the last UV dose, blood samples were collected from the postcaval vein. Values are means ± SD (*n* = 6). ^a,b^ Mean values not sharing the same letter above the bars are significantly different (*p <* 0.05, Tukey’s test). Abbreviations: SM, soymilk; FSM, fermented soymilk; IL-6, interleukin-6.

IL-6 levels in serum were increased by UVB irradiation (*p <* 0.0001, untreated group vs. control group) but were lower in the SM and FSM groups than in the control group (*p <* 0.0001 and *p <* 0.0001, respectively) (Figure 5).

**Figure 6:**
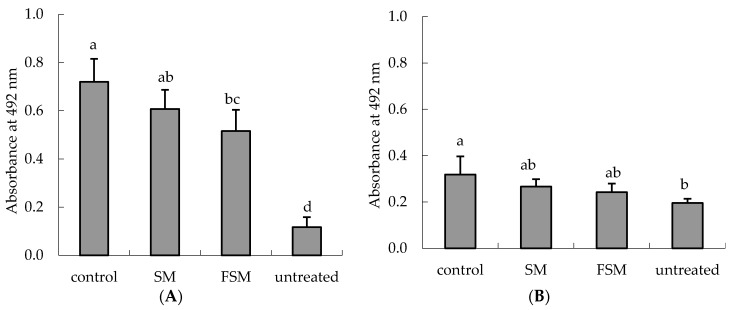
Reduction in ultraviolet (UV) B-induced thymine dimer in genomic DNA in the dorsal skin by the fermented soymilk diet. (**A**) DNA photoproduct cyclobutane pyrimidine dimer (CPD); (**B**) DNA photoproduct (6-4) pyrimidine-pyrimidone photoproduct. The dorsal area was UV irradiated 10 times; 3 h after the last UV dose, dorsal skin was removed and DNA samples were prepared. Values are means ± SD (*n* = 6). ^a,b,c,d^ Mean values not sharing the same letter above the bars are significantly different (*p <* 0.05, Tukey’s test). Abbreviations: SM, soymilk; FSM, fermented soymilk.

The DNA photoproducts CPD and 6-4PP were significantly increased in the dorsal skin by UVB irradiation (*p <* 0.0001 and *p <* 0.0011, respectively). The CPD level was significantly lower in the FSM group than in the control group (*p =* 0.0012) and the level tended to be lower in the SM group than in the control group (*p =* 0.0959) (Figure 6A). The 6-4PP level tended to be lower in the FSM group than in the control group (*p =* 0.0502) (Figure 6B).

There were no differences in the levels of LPO in serum and TBARS in the skin among any of the groups (data not shown).

#### 3.2.2. Bioavailability of Isoflavones

Absorbed isoflavones are mainly present in glucuronide- or sulfate-conjugated forms but are also present in the unconjugated form (free isoflavone) [30,31]. In this study, we measured the levels of free isoflavones and total isoflavones (both free and conjugated) and the following molecular species of isoflavones: genistein, daidzein, glycitein, dihydrodaidzein (DHD), *O*-DMA, and equol (Table 1 and Table 2).

Total isoflavones (any type) were detected in the skin but the DHD concentration was lower than the determination limit (50 pmol/g skin). The concentrations of genistein, daidzein, glycitein, and *O*-DMA, but not equol, in the skin were significantly higher in the FSM group than in the SM group (*p =* 0.044, *p =* 0.029, *p =* 0.041, and *p =* 0.045, respectively). Free isoflavones, DHD, *O*-DMA, and equol were not detected in the skin. The concentrations of genistein, daidzein, and glycitein in the skin were significantly higher in the FSM group than in the SM group (*p =* 0.018, *p =* 0.006, and *p =* 0.002, respectively).

Total isoflavones (any type) were detected in serum, but the DHD concentration was lower than the determination limit (50 nM). The concentrations of genistein and daidzein in serum were significantly higher in the FSM group than in the SM group (*p =* 0.0003 and *p =* 0.004, respectively).

Free isoflavone, DHD, *O*-DMA, and equol were not detected in serum. The concentrations of genistein and daidzein in serum were significantly higher in the FSM group than in the SM group (*p =* 0.0006 and *p =* 0.002, respectively).

## 4. Discussion

Several studies have reported that isoflavones have photoprotective effects, but most studies involved topical application [10,12,13] and few studied the effects of orally administered isoflavones [32]. In addition, few reports have examined the bioavailability of ingested isoflavones. If the isoflavone acts directly, it is necessary to verify whether the isoflavone reaches a target site such as the skin after oral administration. This study aimed, using two types of soymilk with different isoflavone absorption properties (SM and FSM), to investigate the ability of orally administered isoflavones to protect against the inflammation and DNA damage induced by UVB irradiation and furthermore to investigate isoflavone bioavailability. We found that the FSM diet prevented the increase in erythema intensity, epidermal thickness, CPDs, and 6-4PPs in the dorsal skin and in the IL-6 concentration in serum induced by UVB irradiation (Figure 3, Figure 4, Figure 5 and Figure 6). Although some of these protections were also observed with the SM diet, the protective effects were more potent in the FSM diet group (Figure 3 and Figure 4). However, FSM and SM had no effect on the skin (skin thickness and erythema) under the non-UV irradiation condition.

Isoflavone concentrations in the serum and dorsal skin of the FSM diet group were significantly higher than those of the SM diet group (Table 1 and Table 2). We previously reported that the absorption of isoflavones is faster and greater in an aglycone-enriched FSM group than in a glucoside-enriched SM group after a single administration to humans and rats [24,25,27]. Because similar results were obtained in this study, it may be that the serum concentrations of the isoflavones are kept higher by continuous ingestion of aglycone-enriched FSM. However, there was no difference in the concentration of equol between the two groups, which is reported to be the most potent of all isoflavones, although the concentrations of genistein and daidzein in the serum and dorsal skin were higher in the FSM group than in the SM group. Hence, we believe that the higher delivery of genistein and daidzein to the serum and dorsal skin is due to differences in effectiveness between FSM and SM.

Almost all isoflavones are glucuronized and/or sulfated after absorption [30]. However, in this study, about 10% of the total isoflavones in the blood and about 30% of the total isoflavones in the skin were present in the unconjugated aglycone form. The skin may metabolize glucuronized and/or sulfated isoflavones differently.

It is unclear why the results of the unconjugated proportion of isoflavones differed between the skin and the blood. However, Shimoi and co-workers reported that during inflammation, β-glucuronidase is released from stimulated neutrophils or from certain injured cells before deglucuronidation of flavonoids occurs [33,34]. Because we measured isoflavones in the blood and the dorsal skin 3 h after the last dose of UVB irradiation, it may be that the inflammation induced by UVB irradiation stimulated deconjugation of isoflavones in the blood and dorsal skin.

Exposure to UV radiation generates various reactive oxygen species (ROS) within the cell, such as hydrogen peroxide, superoxide anion, and singlet oxygen [35,36]. ROS generated by UV radiation affects cell receptors and their ligands and induces the production of inflammatory cytokines such as IL-1, IL-6, and TNF-α in keratinocytes [37,38]. In addition, these ROS cause lipid peroxidation and DNA and protein damage [39,40]. In this study, the isoflavone concentrations in the blood and skin were kept high by the administration of FSM (Table 1 and Table 2). Therefore, we believe that ROS can be effectively neutralized by isoflavones, leading to the prevention of DNA damage and inflammatory cytokine induction. 

Increase in skin thickness is considered a typical response to protect cells such as keratinocytes in the basal layer of the epidermis and fibroblasts in the dermis after UV irradiation. In this study, FSM administration prevented not only skin thickening but also erythema by attenuating inflammation (IL-6 induction) as well as DNA damage (Figure 3, Figure 4 and Figure 5, Table 1 and Table 2), although there were no significant differences between the two groups in the levels of LPO and TBARS. This may be because the UVB irradiation dose in this study was lower than that in another report [10]. Therefore, these findings indicate that FSM has the potential to prevent skin damage by UV irradiation.

According to the initial UVB irradiation experiments, unlike in ovariectomized mice, there was no influence of the UVB radiation on erythema in the sham-operated mice (Figure 2). Thus, estrogen depletion by ovariectomy might increase the sensitivity of hairless mice to UVB irradiation and erythema as an inflammatory response. These findings support the observation that estrogen insufficiency decreases defenses to oxidative stress [41]. Because of their similar structures, isoflavones exert estrogen-like activity and can protect against hormone-dependent diseases. We believe that the estrogen-like activity of isoflavones from FSM partly compensates for the estrogen depletion in OVX hairless mice, preventing the photodamage induced by UVB irradiation.

Hairless mice are used widely in UV irradiation studies, although their skin is indeed very different from that of humans. It is therefore necessary to consider these differences in studies concerning topical application to the skin. However, our study instead focused on the oral administration route in examining isoflavone bioavailability. We found that orally administered isoflavones reached the target site, namely, the skin, in OVX hairless mice. Thus, we believe that the present model is a good model for the study of bioavailability following oral administration.

## 5. Conclusions

The present study demonstrated that oral administration of an FSM diet maintained high isoflavone concentrations in the skin, suppressing skin photodamage by scavenging ROS generated by UVB irradiation and exerting estrogen-like effects in OVX hairless mice.

## Figures and Tables

**Table 1:** Isoflavone concentrations in the serum of hairless mice.

Isoflavones	Free Isoflavone		Total Isoflavone		Ratio of Free Isoflavone	
	SM Group	FSM Group	SM Group	FSM Group	SM Group	FSM Group
	nM		nM		% (Free/Total)	
Genistein	28.2 ± 17.5 *	73.8 ± 14.5	343.1 ± 187.2 *	1175.7 ± 333.7	8.7 ± 3.6	6.5 ± 1.4
Daidzein	31.3 ± 31.1 *	99.6 ± 27.0	238.2 ± 189.1 *	619.7 ± 171.6	11.0 ± 5.4	16.5 ± 4.7
Glycitein	3.4 ± 2.6	4.0 ± 1.0	76.1 ± 54.6	125.8 ± 39.9	6.3 ± 4.2	3.2 ± 0.6
DHD	nd	nd	<50	<50		
Equol	nd	nd	1100.4 ± 531.7	1429.4 ± 278.3		
*O*-DMA	nd	nd	394.9 ± 377.3	255.6 ± 90.9		

Values are means ± SD (*n* = 6). * *p <* 0.05 indicates significant differences in isoflavone concentrations between the SM and FSM groups (unpaired *t*-test). Abbreviations: SM, soymilk; FSM, fermented soymilk; DHD, dihydrodaidzein; *O*-DMA, *O*-desmethylangolensin; nd, not detected.

**Table 2:** Isoflavone concentrations in the dorsal skin of hairless mice.

Isoflavones	Free Isoflavone		Total Isoflavone		Ratio of Free Isoflavone	
	SM Group	FSM Group	SM Group	FSM Group	SM Group	FSM Group
	pmol/g Skin		pmol/g Skin		% (Free/Total)	
Genistein	121.5 ± 28.1 *	204.7 ± 66.2	336.7 ± 170.2 *	691.1 ± 335.5	39.5 ± 8.8	31.1 ± 4.3
Daidzein	38.9 ± 8.8 *	158.5 ± 84.1	110.0 ± 27.7 *	509.0 ± 381.3	35.8 ± 3.2	34.3 ± 7.7
Glycitein	14.3 ± 4.1 *	46.1 ± 18.5	43.3 ± 11.3	196.4 ± 159.8	33.0 ± 2.3	29.8 ± 14.9
DHD	nd	nd	<50	<50		
Equol	nd	nd	313.3 ± 49.6	398.5 ± 111.2		
*O*-DMA	nd	nd	106.4 ± 63.0	244.2 ± 133.6		

Values are means ± SD (*n* = 6). * *p <* 0.05 indicates significant differences in isoflavone concentrations between the SM and FSM groups (unpaired *t*-test). Abbreviations: SM, soymilk; FSM, fermented soymilk; DHD, dihydrodaidzein; *O*-DMA, *O*-desmethylangolensin; nd, not detected.

## Supplementary Materials

The following are available online at http://www.mdpi.com/2072-6643/8/8/514/s1, Table S1: Composition of isoflavones in soymilk and fermented soymilk powder; Table S2: Compositions of the experimental diets; Table S3: Body weight and intake of food in hairless mice in the untreated group (fed the control diet), the control group (fed the control diet), the SM group (fed the soymilk diet), and the FSM group (fed the fermented soymilk diet).

## Abbreviations

The following abbreviations are used in this manuscript:

SM	Soymilk
FSM	Fermented soymilk
OVX	Ovariectomized
TBARS	Thiobarbituric acid-reactive substances
UVB	Ultraviolet B
*O*-DMA	*O*-Desmethylangolensin
LPO	Lipid peroxide
CPD	Cyclobutane pyrimidine dimer
ROS	Reactive oxygen species
6-4PPs	Pyrimidine-pyrimidone photoproducts
DHD	Dihydrodaidzein
