# Supplementary material for: Oral Administration of Fermented Soymilk Products Protects the Skin of Hairless Mice against Ultraviolet Damage
Source: Nutrients. 2016 Aug 20;8(8):514. doi: 10.3390/nu8080514 (PMC4997427; doi:10.3390/nu8080514)
Supplement: Supplementary file 1 [file nutrients-08-00514-s001.docx]

**Supplementary Materials: Oral Administration of Fermented Soymilk Products Protects the Skin of Hairless Mice against Ultraviolet Damage**

Mitsuyoshi Kano, Norihiro Kubota, Norie Masuoka, Tetsuji Hori, Kouji Miyazaki and Fumiyasu Ishikawa

**Table S1.** Composition of isoflavones in soymilk and fermented soymilk powder.

| **Isoflavone** | **Soymilk** | | **Fermented Soymilk** | |
| --- | --- | --- | --- | --- |
|  | **mg/g (μmoL/g)** | | **mg/g (μmoL/g)** | |
| Daidzin | 2.198 | (5.278) | 0.001 | (0.002) |
| Genistin | 1.870 | (4.325) | 0.000 | (0.001) |
| Glycitin | 0.460 | (1.030) | 0.000 | (0.000) |
| Malonyl daidzin | 0.298 | (0.592) | 0.001 | (0.003) |
| Malonyl genistin | 0.601 | (1.159) | 0.002 | (0.004) |
| Malonyl glycitin | 0.266 | (0.499) | 0.001 | (0.002) |
| Daidzein | 0.017 | (0.065) | 1.280 | (5.035) |
| Genistein | 0.008 | (0.030) | 1.317 | (4.874) |
| Glycitein | 0.004 | (0.014) | 0.421 | (1.483) |
| Total | 5.722 | (12.992) | 3.023 | (11.404) |

**Table S2.** Compositions of the experimental diets.

|  | **Control Diet** | **SM Diet** | **FSM Diet** |
| --- | --- | --- | --- |
|  | **g/kg Diet** | | |
| α-Cornstarch | 397.5 | 397.5 | 397.5 |
| Casein | 200.0 | 112.3 | 112.3 |
| Dextrinized cornstarch | 132.0 | 132.0 | 132.0 |
| Sucrose | 100.0 | 41.2 | 41.2 |
| Corn oil | 70.0 | 16.5 | 16.5 |
| Cellulose powder | 50.0 | 50.0 | 50.0 |
| Mineral mixture (AIN-93G-MX) | 35.0 | 35.0 | 35.0 |
| Vitamin mixture (AIN-93-VX) | 10.0 | 10.0 | 10.0 |
| l-Cystine | 3.0 | 3.0 | 3.0 |
| Choline bitartrate | 2.5 | 2.5 | 2.5 |
| Soymilk powder ^1^ | 0.0 | 200.0 | 0.0 |
| Fermented soymilk powder ^2^ | 0.0 | 0.0 | 200.0 |

^1^ Protein, 40.18%; fat, 28.57%; ash, 6.25%; fiber, 0%; carbohydrate, 25%; ^2^ Protein, 41.28%; fat, 30.28%; ash, 5.5%; fiber, 0%; carbohydrate, 22.94%; Abbreviations: SM, soymilk; FSM, fermented soymilk.

**Table S3.** Body weight and intake of food in hairless mice in the untreated group (fed the control diet), the control group (fed the control diet), the SM group (fed the soymilk diet), and the FSM group (fed the fermented soymilk diet).

|  | **Untreated Group** | **Control Group** | **SM Group** | **FSM Group** |
| --- | --- | --- | --- | --- |
| Initial body weight, g | 19.5 ± 0.5 | 20.0 ± 1.2 | 19.3 ± 1.1 | 19.8 ± 0.9 |
| Final body weight, g | 24.3 ± 1.0 | 24.7 ± 1.9 | 24.0 ± 1.9 | 24.5 ± 1.4 |
| Food intake, g/day | 4.3 ± 0.5 | 4.1 ± 0.7 | 4.4 ± 0.3 | 4.6 ± 0.3 |

Values are means ± SD (*n* = 6); Abbreviations: SM, soymilk; FSM, fermented soymilk.
